# Waxy Kidney—with Case

**Published:** 1874-05

**Authors:** M. W. Wood


ARTICLE II.—*Waxy Kidney—With Case.* Read before the Chicago Society of Physicians and Surgeons, December 8, 1873. By M. W. WOOD, M.D.

Late in the first quarter of the present century, a discovery was made which marked an era in the history of medicine; I allude to the results of the researches of Dr. Richard Bright, which were first published in London, in 1827, in the first volume of his "Reports of Medical Cases."

Unsatisfied with the little which was known of the real pathology of certain forms of dropsy associated with albumen in the urine, he improved the advantage of his position as physician to Guy's Hospital, and made the investigations which have had the result of giving the name of "Morbus Brightii" to all the various forms of degenerative processes which have been discovered in the kidney.

The different pathological states which are included in this general class are quite various; differing in modes of origin, in morbid anatomy, in symptoms, and in course; and as to where the line should be drawn, between these and other pathological conditions of the kidney not to be thus included, pathologists are undecided; even those who would most sharply make such distinction, differ in the conditions to be included. There is, however, tolerable uniformity existing with regard to the inclusion of an inflammatory form, one of fatty degeneration, one of amyloid or waxy degeneration, and one of cirrhosis.

It being impracticable, within the limits of this paper, to treat of "Bright's Diseases" in general, I shall confine myself to a consideration of some of the more prominent points in the least common of the essentially different morbid processes generally recognized as included under this general term, *i. e.*, the amyloid or waxy degeneration, or, as Niemeyer* defines it, "parenchymatous nephritis, with amyloid degeneration of the walls of the vessels, for very unusually is the tunica propria, and still more rarely are the epithelial cells affected." Johnson, an eminent authority upon this general subject, says,† "all of the changes of structure commence in the secreting cells of the gland, and are the results

* Niemeyer. Text Book of Practical Medicine.

† Johnson. Diseases of the Kidney. 1st Edition. London, 1852.

of an effort made by the cells to eliminate from the blood some abnormal products—some materials which do not naturally enter into the composition of the renal secretion." Stewart, another high authority, divides the condition into three stages: of which he terms the first, one of degeneration of the vessels; the second one of secondary changes in the tubes; and the third, one of atrophy.*

A careful consideration of the subject, however, in the light which general pathology brings to bear upon it, causes a dissent from these views, and forces us to the conclusion that while the normal epithelium still lines the tubules, the protoplasm thrown out is, in obedience to the laws of nutrition, built up into tissues or secretion like those which preceded it; but, the cell-type once lost, the exudate which is being continually thrown out, having now no physiological function to perform, becomes degenerate, and the particular form of degeneration which occurs is that to which the exudate was predisposed.†

True, *atrophy*, *i. e.*, a small, contracted, granular kidney closes the scene in this, as in each of the other forms of "Bright's Disease," though each form is characterized by different anatomical changes; but this fact, though stated generally in the course of their essays, seems to have been lost sight of by our authors in making up their list of deductions, else we would not have had a "Gull-Sutton-Johnson controversy."

The nature of this substance, the result of the degeneration has been variously regarded; thus, Dickinson‡ considers it a variety of fibrin, and Sir Wm. Gull§ speaks of it as a "hyalin fibroid" substance; Roberts says,‖ "to call it amyloid is simply a misnomer, and an unfortunate one, as it leads to confused notions as to the existence of some connection between waxy degeneration and the (genuine) amyloid substance found in the healthy liver. * * * * That it is essentially a degeneration, and in no sense an infiltration by any ingredient of the blood, becomes apparent on consideration of the parts affected."

* Stewart. Bright's Diseases. 2d Edition. N. Y., 1872.

† Prof. J. Adams Allen. Lectures on General Pathology. Chicago, 1872–3.

‡ Roberts. Urinary and Renal Diseases. 2d Edition. Philadelphia, 1872.

§ Proc. Royal Med. Chi. Soc. Med. Times and Gazette. London, November, 1872.

‖ Op. cit.

The waxy kidney occurs chiefly in persons of a strumous diathesis; and more often in males than in females, because they are more exposed to its exciting causes; for the same reason, adults are more particularly liable than children; and, of adult males, those whose occupation is one of exposure to wet and cold.

The disease is generally symmetrical; that is, both kidneys are more or less affected, though the disease is frequently more advanced in one kidney than in the other; cases, nevertheless, have been reported, where one kidney was in an advanced state of degeneration, and its fellow normal;* in such cases, however, there is albuminuria, but no dropsy.†

The symptomatology of this disease merits our careful attention, but a consideration in detail of the various symptoms would be too tedious, and a few, only, of the more prominent ones will be presented; noticing, however, in passing, that its insidious approach, steady progression, and probable duration, are pointed out by various morbid appearances, which also give us valuable guides as to treatment.

*The Anasarca.*—This, which led Dr. Bright to his important discoveries, is generally the first to induce the patient to seek advice, and directs attention to the real trouble, as in the case which I am about to relate. The rate of development of the anasarca may, as Johnson says,‡ "be at any time measured by the rapidity with which the imprint of the finger is effaced from the edematous surface;" an observation which will be borne out by the testimony of those who have carefully observed this phenomenon. The anasarca seems to depend upon:

*a.* The amount of destruction of gland-tissue, as anasarca makes its appearance only when there is no longer sufficient kidney structure intact to separate the urinary excrementitious substances.§

*b.* The amount, and rapidity of accumulation of these substances in the blood.

*c.* The density of the blood-serum; and here it may be noticed that there is a definite inverse ratio between the coagulability of the urine and this density.

* London Path. Soc.'s Trans., Vol. 19.

† Harley. The Urine and its Derangements. Philadelphia, 1872.

‡ Op. cit.

§ Harley, op. cit.

*d.* The amount of capillary obstruction; and it should be borne in mind that the flow of blood through the capillaries is to a certain extent affected by the composition of the blood itself; it being a well known principle of physics that fluids pass readily through capillary tubes for the walls of which they have an affinity.*

*e.* The amount of urine voided; it being obvious that the danger of anasarca is lessened in proportion to the amount of diuresis.

*The Appearance of the Patient.*—Together with the pasty look of the face, there is a pallid or dingy hue of the skin, of which Dr. Loomis remarks:† "It is peculiar; not like the clear pallor of phthisis, nor the dingy pallor of cancer; not easily described, but peculiar to 'Bright's Disease,' and easily recognized after it is once seen." Stewart‡ also calls attention to a peculiar characteristic appearance of the face, which he has observed, "when the surface generally is pale and clear, but a very distinct congestion exists over the cheeks; not a congestion like a blush, but one which is seen by the naked eye to depend upon the distension of small vessels, quite above the size of capillaries."

*The Urine.*—Albumen is the most common and most important of the abnormal ingredients of the urine, and though it occurs in various diseases, its presence, aside from "Bright's Diseases," is transient, and the quantity small. When we consider the fact that of all animal substances, albumen possesses the greatest endosmotic power, and must, together with the water and salts, pass from the malpighian capillaries, it seems somewhat strange that it does not appear as a constituent of normal urine; but in its passage through the tubule, it must subserve some further nutritive process, or receive some transformation by contact, either with the cells of the tubule, or with the solid excrementitious portions of the urine which are separated from the inspissated blood of the second set of capillaries.§

Of the urinary constituents, urea is also of great importance, as a measure of the activity of the general process of disassimilation—the wear and tear of the system.‖ Since its discovery in 1771, by Hillaire Rouelle, there have appeared in our literature numerous

* Prof. J. Adams Allen, op. cit.

† Prof. Alfred L. Loomis. N. Y. Med. Record, May 1st and 15th, 1873.

‡ Op. cit.

§ Vide Harley, op. cit.

‖ Harley, op. cit.

and valuable observations upon this subject, with this conclusion as a general result: that urea, though found in the urine, is not formed by the kidneys, but that those organs merely excrete it; that it is not the special product of any particular substance or organ, but one of the products of the retrograde metamorphosis of all nitrogenized tissues.* In the diagnosis of this morbid affection, there are to be taken into consideration:

*a.* The mode of invasion; was it insidious.

*b.* The condition at onset; as this form never occurs as a primary disease in otherwise healthy persons, but as a sequel always.

*c.* The etiology; as if it be fairly due to syphilis, to scrofula, to long continued suppuration, to caries or necrosis, or to any other wasting malady, it is presumably waxy.

*d.* The prominent symptoms; as if polyuria has existed from the beginning, we should, judging from this alone, be compelled to class it as waxy. Of this symptom, a distinguished author remarks:† "it is an unfailing symptom at the early stage in the waxy form, and is absent only when inflammation becomes superadded, or diarrhœa is draining the blood of fluid."

*e.* The existing complications; the most common concomitant affections being a similar degeneration of the liver and spleen.

The great difficulty in diagnosis, however, is not so much in determining the form, as the stage; and for this we must depend upon our knowledge of the anatomical changes which are taking place in the kidney, as determined by:

*a.* The color and amount of the urine.

*b.* The amount of albumen; which early is scanty, later abundant, and still later again scanty.

*c.* The sediment; and its character as revealed by the microscope.

*d.* The size of the casts; it being evident that the cast formed in a tube which has been deprived of its epithelium, is larger than one from a tube not so denuded.

Of the importance of microscopy in this disease, Aitken says,‡ "without a microscopic examination of the urine from day to day, it is impossible to distinguish between a case likely to improve under treatment and one that may be regarded as hopeless, and

* Harley, op. cit. † Stewart, op. cit.

‡ Science and Practice of Medicine. 4th Edition, Vol. 2, p. 163.

without the daily use of the microscope, treatment becomes at the best but guesswork."

The pathology generally received is: that there is an amyloid degeneration occupying the place of the tubal epithelium; an amyloid or "*hyalin fibroid*"* degeneration of the walls of the blood-vessels, as follows:

1. The intertubular, or second set of capillaries.
2. The intercapillary vessels, or venules.
3. The malpighian capillaries, or capilliculi.
4. The afferent vessels.

Thence extending around the convoluted tubes, and malpighian tufts: that this substance subsequently contracts, and draws the malpighian bodies together; compresses the uriniferous tubules, and may entirely obliterate them: that this compression interferes with nutrition and tends to atrophy. And some pathologists regard this amyloid matter as a source of irritation, causing proliferation of the intertubular connective tissue,† and thus the production of a cirrhotic kidney, at the close of a history of waxy degeneration.

Of the complications most commonly presented, we learn from Stewart,‡ who has statistics of a large number of cases, that waxy degeneration of the liver exists in from seventy to eighty per cent. of the cases; waxy degeneration of the spleen in from seventy to eighty per cent., (Roberts§ says he found it in forty-eight out of seventy-seven cases); waxy degeneration of the alimentary tract in about fifty per cent.; pulmonary tuberculosis in about one-half of the cases; caries and necrosis in from ten to fifteen per cent., and that diarrhœa from vicarious elimination is quite common. The prognosis may be summed up in a single word—unfavorable.

The treatment of renal structural disease in any form, has not long been regarded as very promising; but increased powers of diagnosis, and the great advance made in the knowledge of their pathology have at least enabled us to separate those that we may regard as curable, from those the severity of which can at best be but mitigated. To be at all successful in treatment we must be correct in diagnosis; and not, having found a name for the disease, cease our endeavors to ascertain its cause. As we cannot, unfor-

* Proc. Royal Med. Chi. Soc. London, November, 1872.

† Phil. Med. Times, March 22d, 1873.

‡ Op. cit. § Op. cit.

tunately, procure physiological rest for these organs, we endeavor to make their task an easier one, by lessening the amount of labor to be performed, and inducing vicarious action of the other emunctories, and thereby at least gain time, which often becomes an important element of treatment. The perspiration, even in health, contains urea, chlorides, phosphates and sulphates;* and in disease even the insoluble oxalate of lime is sometimes found in considerable abundance.† The urinous odor of the perspiration and breath has been often noted, and urea found in considerable abundance in the intestinal evacuations; it may appear, says Harley,‡ "in all of the fluids of the body." Thus, as elsewhere, the teachings of physiology furnish us with a key to the proper mode of treatment. In this condition, the indications for treatment obviously are:

*a.* To check, if possible, the progress of the degeneration;

*b.* To prevent the addition of complications, particularly inflammatory ones; and

*c.* To palliate, as far as possible, the more troublesome symptoms.

And these indications we seek to fulfill by measures therapeutic, dietetic, and prophylactic.

The therapeutics seems at best but doubtful; remedies we doubtless have, but we lack the knowledge and skill to apply them, "no medicine having yet been discovered which would produce any direct effect upon the waxy degeneration itself."§

The first of these indications can be but poorly met. If we remove from the tubes, by any means, the obstructive exudate, which is constantly provoking degenerative inflammation, and hindering elimination by preventing secretion, we do all that we can hope to do, and for this purpose we have a remedy, digitalis, which will increase the secretion without stimulating the kidney,‖ as it will render the heart's movements more strong, energetic and regular, augment the arterial tension, increase the fullness and force of the pulse, contract the arteries and arterioles, and moderate the number of pulse beats in direct proportion to the elevation of

* Austin Flint, Jr. Physiology, 3d Volume. N. Y., 1870.

† Harley, op. cit.

‡ Op. cit.

§ Stewart, op. cit.

‖ Loomis, op. cit.

arterial pressure;* bearing in mind that digitalis has no influence upon the lamina of normal vessels.† This remedy was the one originally recommended by the distinguished discoverer of the malady, who gave it in combination with the bitartrate of potassa. It may be given in infusion, in small doses, often repeated, until its effects are produced; then in larger doses, at longer intervals, to continue the effect.

In belladonna we have a powerful adjuvant to the remedy already considered; it does not lower the temperature as digitalis does,‡ but it increases the contractions of the heart and arteries and produces an increase of temperature throughout the body, without increasing the respirations, or interfering with them, save in the production of an occasional yawn or long-drawn sigh; this phenomenon in the case of my patient so alarmed his mother that she sent for me with a message that he was dying, at a time when he was comparatively quite comfortable. This medicine is rapidly eliminated by the kidneys, increasing the elimination of urea as well as of the phosphates and sulphates; but the urea is increased out of proportion. It has, besides, a direct effect upon the amount of albumen in the urine; Stewart§ says, "as soon as the constitutional effect appeared, the albumen diminished, and in the course of a month it could be discerned only by careful testing; that this was really due to the medicine, and not to the natural progress of the malady, was proved by the fact that, the medicine being suspended for a week, about the middle of the time the albumen increased."

In elaterium, when it is not contra-indicated, we have another powerful aid to the fulfillment of this indication, as it is eliminative of the urea, which has been found in abundance in the stools produced by it.‖ These stools are copious and watery, and have been well compared to water in which meat has been partially boiled.¶ Colchicum and guaiacum are also powerful eliminatives of urea.**

* Dr. Gourval. Gazette Medicale, December 30, 1871. In New Remedies for April, 1872.

† Dr. Rudolph Boehm. Pfluger's Archiv., Feb., 1872. In New Remedies for July, 1872.

‡ Waring. Therapeutics.

§ Op. cit.

‖ Bird. Urinary Deposits. 5th Ed.

¶ Waring. Op. cit. ** Bedford. Diseases of Women and Children.

The second indication is to be met by prophylaxis chiefly; the patient should remain in bed, in a temperature moderate and uniform; especially is this necessary during cold weather, when careful watching to prevent exposure to sudden draughts of cold air, is also necessary.

Flannel should be worn next the skin. The diet should be plain, easily digestible, nutritious, and unstimulating; in most cases, also, it will be proper to abstain from sugar;* tea and coffee without sugar may very properly be allowed, as both increase the excretion of urea, while alcoholics and beer should be avoided, as their effect is just the reverse of this.†

Great thirst is frequently complained of, and is satisfied, if at all, only by large draughts; and if it be first carefully explained that over-indulgence will produce unpleasant effects, the patient may generally be allowed to use his own discretion in the matter of drink; as, says Watson,‡ "he is better able than his physician to judge which evil is the greater—the torment of unslaked thirst, or the misery produced by over-indulgence."

The third indication is to be met chiefly by means already pointed out; for the treatment of the ascites so often present in these cases, and probably dependent upon the hepatic complication, elaterium offers a greater prospect of success than any other known remedy; though serviceable in other dropsies, it is particularly so in this; Stille§ says, "in cases of abdominal dropsy which resist these remedies [referring to diuretics], as too many will do, a more certain resource is offered by elaterium."

The anemia calls for the administration of iron, and of the ferruginous preparations the tincture of the chloride seems the most eligible, as, besides, it checks the drain of albumen.

I will now briefly recount the history of a case, from my notes taken at the time:

*Case.* H—— M——, aged 18 years; born in Buffalo, N. Y., of Irish parentage; first saw the case October 31st, 1873. Prior to present illness had been generally healthy, though of a marked scro ulous diathesis, as are all the other members of the family, *i. e.*, widowed mother and three other children.

* Prout. Stomach and Renal Diseases. 5th Ed.

† Harley. Op. cit.

‡ Practice of Physic. 4th Ed. § Therapeutics and Materia Medica.

In the summer of 1866, while at play, he leaped to the ground from the roof of a one-story school-house; was stunned, as might have been expected. For some time afterward nothing in particular was noticed, except that he had become deliberate in his movements, and soon he began to drag his feet as he walked, or shuffled along. Polyuria existed from the date of the injury. He has, during the seven years which have since elapsed, been under treatment continuously by various physicians of all schools. Has, as his mother says, been treated *principally* for: big liver; big kidneys; worms; *dropsy;* broken back; and abscesses, of which he has two, one discharging on the inner aspect of either thigh.

It appears that a short time (?) after the injury, a lumbar abscess proper (*i. e.*, in the posterior chamber of the lumbar fascia) was noticed, which ran its course; later the two psoas abscesses appeared almost simultaneously, and have discharged continuously ever since, during which time the bodies of *some* of the dorsal vertebræ became involved, and a very decided anterior curvature has resulted from the Potts disease.

On examination he presents a leuco-phlegmatic aspect, and the peculiar pallor of which I have spoken; general nutrition of body poor, very much emaciated; in bed, in a sitting posture, unable to assume any other on account of the orthopnœa, which is very troublesome; is anasarcous markedly, and, besides, has ascites; abdomen much distended, smooth, white, and shining; temperature 96½° F. (morning temperature) and apparently cooler; skin dry; pulse 90, irregular, small, and weak; respiration 18, and normal in character, if the position be not taken into account; appetite very good, eats almost anything, and appears to enjoy his food; thirst not at all excessive; bowels a little loose but regular; stools normal in appearance, but smelling very badly; urination not at all painful; amount passed in 24 hours, about 20 oz., sp. gr. 1011½, of a pale straw color; the natural urinous odor replaced by a faint, unpleasant one; albuminous, the coagulum being one-fourth the height of the quantity tested; on microscopic examination the urine was found loaded with uric acid and phosphates, amorphous and crystallized; sediment consisting largely of large, pale, hyalin casts, averaging in diameter about 1-1800 in. (camera lucida); some disintegrated *tubal* epithelium, a few disintegrated blood corpuscles, together with the uric acid and phosphates already alluded to. The patient, aside from the orthopnœa (avoided by position), and the annoyance caused by the discharge from the abscesses, seemed quite comfortable, not at all restless, somewhat drowsy, but generally cheerful and contented.

No particular pain was complained of. Paralysis of both lower extremities was complete, showing that the spinal cord had become involved; the bladder and rectum were, however, uninterfered with. The heart was found somewhat hypertrophied, but gave no further evidence of disease. The liver was found slightly enlarged; its lower border and surface nodulated. The area of splenic dullness was increased, but, owing to the ascites, its size could not be accurately made out. I regretted that I could not avail myself of the means suggested by Dr. Stewart* for diagnosing the splenic degeneration. Resonance on percussion over both lungs was normal, and auscultation gave no evidence of disease. The penis and scrotum were œdematous.

My diagnosis was: Waxy kidneys, liver, and spleen; consequent upon the prolonged suppuration from which he had suffered; and that the concussion was the exciting cause of the first manifestations of morbid action, to which the others were but sequelæ.

I gave him a mixture composed as follows:

| | | |
|---|---|---|
| R. | Fl. Ext. Belladonnæ, . . . . | m. xxjv., |
| | Fl. Ext. Colchici Rad., . . . . | f. dr. jss., |
| | Tinctura Digitalis, . . . | f. dr. iijss., |
| | Syr. Aurantii Cort. Dulc., . | ad f. oz. ij., |
| M. Sig.: | Teaspoonful ter in die. | |

Under the influence of which the general dropsy disappeared, save from the ankles, feet, and genital organs, the ascites partially remaining; his condition was now rendered more endurable, as he could lie on his back or on either side at will. The bowels became a little more loose, and his condition seemed improved. I say seemed, because it was apparent that the powers of life were nearly gone; he sank gradually, and died on the morning of November 28th, 1873. An autopsy was persistently refused. This I regretted very much, as I wished and expected to present

* Lecture on Waxy Kidney. Med. Times and Gazette, London, June 13th, 1873.

the specimens, having neglected to make either drawings or permanent preparations of the urinary sediment, or a careful quantitative analysis of the urine.

